# The Curious Case of a Painful Leg Ulcer

**DOI:** 10.7759/cureus.54127

**Published:** 2024-02-13

**Authors:** Jay R Patel, Rubi Montejano, Christina Hopkins, Hanna Siatecka, Theodore Rosen

**Affiliations:** 1 Dermatology, Baylor College of Medicine, Houston, USA; 2 Pathology and Immunology, Baylor College of Medicine, Houston, USA; 3 Dermatology, Michael E. DeBakey Veterans Affairs Medical Center, Baylor College of Medicine, Houston, USA

**Keywords:** derm path, non-malignant hematology, hydroxyurea use, non-healing ulcer, sickle cell disease complications

## Abstract

Sickle cell disease is a condition that can involve numerous organ systems secondary to vascular occlusion. Herein, we present a case of a 21-year-old male with sickle cell disease requiring long-term hydroxyurea therapy. Upon migrating to the United States from Yemen, the patient developed a rapidly progressive, exquisitely painful ulcer on his right lower extremity. Given his country of origin, a broad differential, including select infectious diseases, was essential. Moreover, establishing the unequivocally correct diagnosis was crucial to determine proper and safe therapy. Ultimately, a lesional biopsy demonstrated numerous sickled red blood cells occluding blood vessels, leading to the diagnosis of sickle cell disease-related leg ulceration.

## Introduction

With its protean multisystem sequelae, sickle cell disease is challenging to treat. When an ulcer appears on the lower extremities, many potential diagnoses may be suggested. In the setting of sickle cell disease, however, two specific causes should always be considered: an ischemic ulcer related to the underlying hematological abnormality and an ulcer caused by hydroxyurea, the most common, chronic therapeutic intervention. We present a case of a patient with a debilitating leg ulcer whose differential diagnosis was further complicated by a distinct infectious possibility associated with his native nationality.

## Case presentation

**Figure 1:**
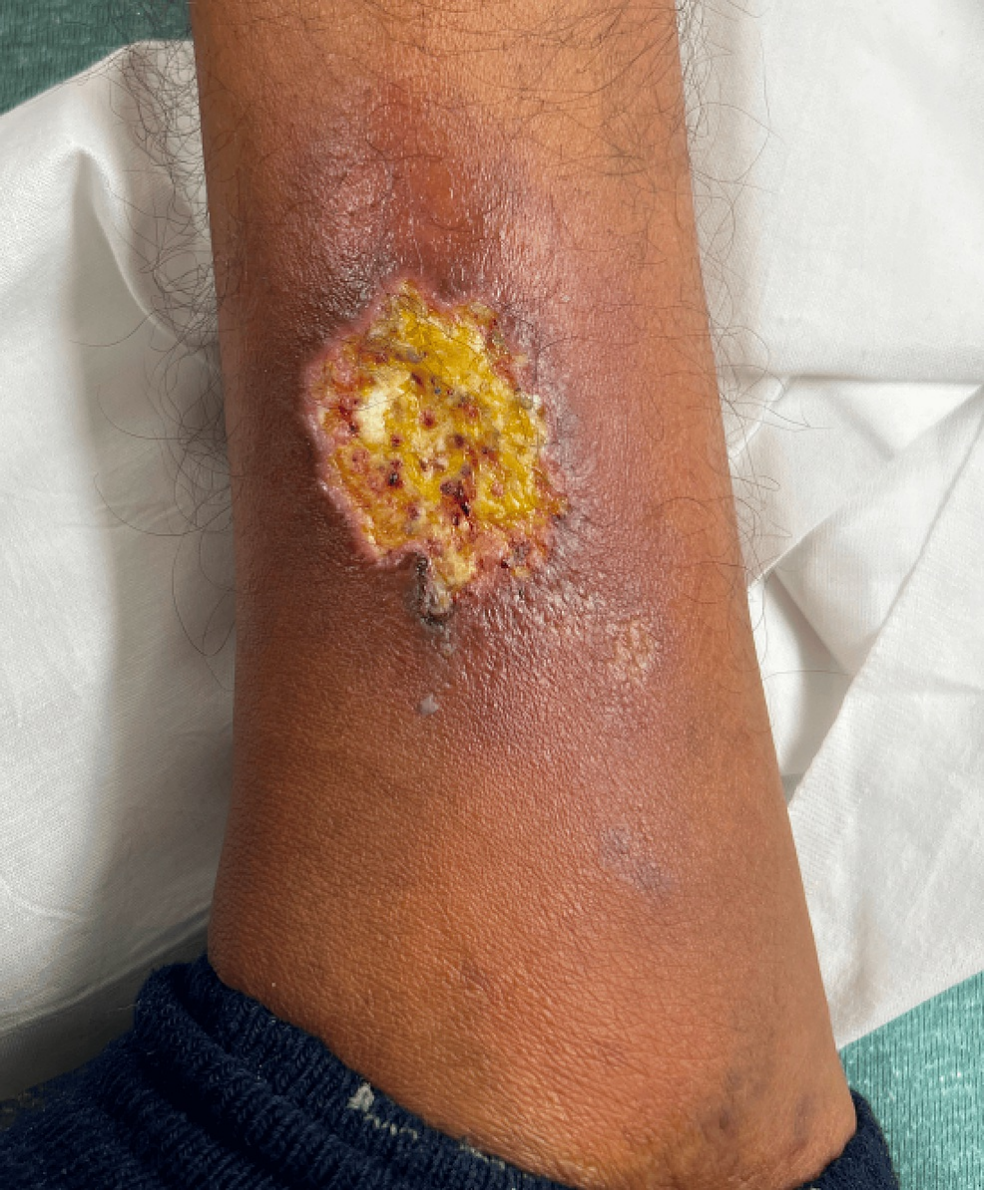
Right medial lower leg with a round, indurated ulcerated nodule with yellow slough and pinpoint areas of hemorrhage

A 21-year-old male with known sickle cell disease, on hydroxyurea and folic acid for the past eight years, presented to the emergency department for an enlarging, extremely painful ulcer on his right lower extremity. The ulcer began two weeks prior to presentation (Figure 1).

**Figure 2:**
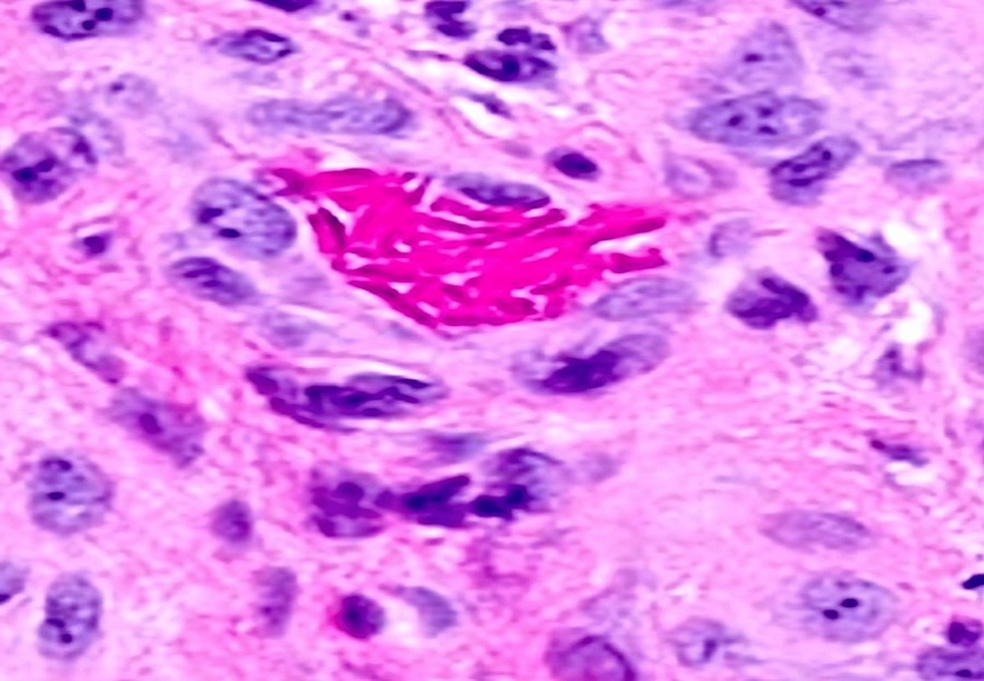
Histopathology of a small-caliber blood vessel occluded by numerous sickle-shaped red blood cells

**Figure 3:**
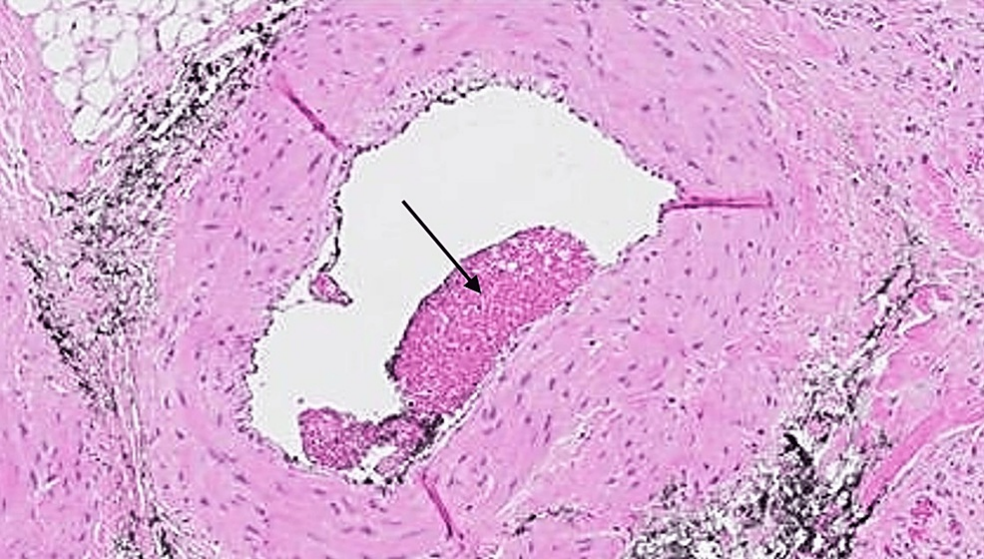
A larger caliber, thicker-walled dermal vessel with partial thrombus (black arrow)

**Figure 4:**
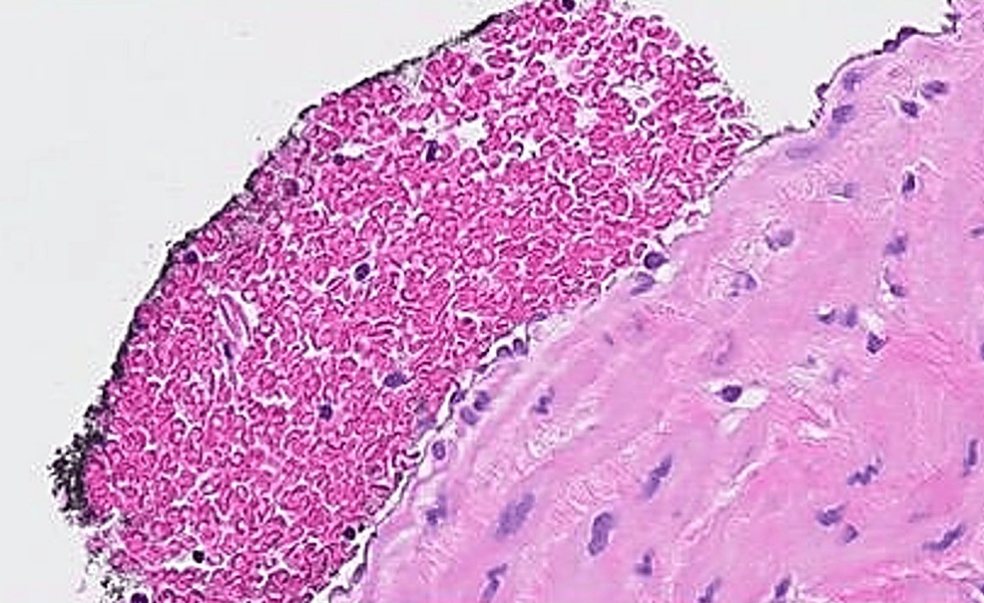
The thrombus found within a large caliber vessel is composed of densely packed, grossly sickle-cell-shaped red blood cells

The patient had moved to the United States from Yemen one week prior. He had applied topical fusidic acid (a common antibacterial used outside of the United States) without improvement. Admission laboratories of note were notable for moderate anemia and otherwise normal renal, hepatic, and other metabolic parameters. Infectious Disease (ID), Hematology, and Dermatology Services were consulted. ID expressed significant concern for the diagnosis of leishmaniasis, given the high prevalence of Old World leishmaniasis in Yemen. Other differential diagnoses included hydroxyurea-induced leg ulcer and sickle cell-mediated ulcer. As proper treatments for these three possibilities are entirely contradictory, Dermatology suggested that a biopsy could potentially settle the diagnostic dispute, thereby leading to the timely institution of correct management. A punch biopsy revealed no indication of infection (specifically an absence of parasitized histiocytes). The biopsy did reveal epidermal ulceration with prominent vascular occlusion of the small and medium-sized dermal vasculature with sickled red blood cells (Figures 2, 3, 4). A perivascular infiltrate was also noted throughout.

The histology allowed the conclusion that the patient was suffering from a sickle cell disease-mediated leg ulcer, rather than an ulcer due to leishmaniasis or an ulcer related to his hydroxyurea therapy per se. The treatment plan included the administration of analgesia, increased hydration, increased dose of hydroxyurea, and possible institution of other therapeutic interventions. The patient did well at the time of discharge but was subsequently lost to follow-up.

## Discussion

Sickle cell disease is a genetic disorder that can be inherited in a variety of ways. The disease manifestations result from the production of abnormal hemoglobin, which, in turn, results in red blood cells that assume a sickle configuration. These abnormally shaped cells occlude dermal vasculature, leading to acute and chronic damage of various tissues [1]. Sickle cell leg ulcers have varying rates of occurrence, with the United States having between 8% and 10% [2-4]. These typically painful, progressive ulcers often occur spontaneously or after localized trauma. Risk factors include infection, venous insufficiency, hemolysis, specific variants of sickle cell disease, baseline hemoglobin, lower socioeconomic status, and multiple chronic transfusions [2-4]. As sickle cell leg ulcers are not common, it is important to keep this entity in mind, as it can closely mimic conditions such as malignancy, infectious (i.e., leishmaniasis, sporotrichosis, atypical mycobacteria), and primary cutaneous conditions (pyoderma gangrenosum, necrobiosis lipoidica) [5].

Leg ulcers primarily occur in areas with little subcutaneous fat. While vaso-occlusion and subsequent infarction and necrosis play a key role in etiopathogenesis, an additional factor includes an intrinsically lower level of nitric oxide, an important vasodilator, in patients with sickle cell disease, potentiating both ischemia and infarction [4]. Hydroxyurea is used as an inexpensive treatment because it promotes the formation of fetal hemoglobin and lessens the risk of red blood cell sickling. However, hydroxyurea has been implicated as a possible cause of leg ulcers. This association is tenuous at best, as illustrated by a meta-analysis that found no correlation; another study demonstrated no difference in the rate of ulcer development with or without the use of therapeutic hydroxyurea [4,6,7]. If a leg ulcer is due to hydroxyurea, histology will show more chronic changes with atrophy, fibrosis, and scarring with far less inflammation and fewer (if any) occluded blood vessels. Sickle cell-mediated leg ulcers will demonstrate more lymphoplasmacytic inflammation and show characteristic vascular occlusion by sickle cells [8,9]. In this case, a skin biopsy was essential to determine the diagnosis and prevent a treatment plan that might have been ineffective or even deleterious.

Sickle cell leg ulcers heal slowly, and there is no consensus on the best way to treat them. The primary therapeutic methods are adequate pain control, wound dressings, and infection prevention [4]. Numerous options have been tried with varying success, such as various topical agents (triple antibiotic ointment and sodium nitrite); a variety of systemic agents (arginine butyrate, zinc, L-carnitine, hyperbaric oxygen therapy); cell-based therapy (pinch graft, hematopoietic stem cell transplantation); and pressure-based treatments (compression stockings, negative-pressure wound therapy) [2]. Prevention of leg ulcers is key and this can be done by optimizing the medical management of sickle cell disease. Hydroxyurea is the mainstay of treatment, but other Food and Drug Administration (FDA)-approved options include L-glutamine, crizanlizumab, and voxelotor. Of note, the FDA also recently approved two genetic-based treatments, lovotibeglogene autotemcel and exagamglogene autotemcel, which permanently alter the hemoglobin produced. Unfortunately, these genetic therapies currently cost $2-3 million dollars. These treatments, along with others being developed, will hopefully improve management options for sickle cell disease and, as a consequence, improve treatment options for sickle cell disease-related ulcers.

## Conclusions

Sickle cell disease can rarely manifest with leg ulcers, most commonly on the lower extremities. Numerous other etiologies can mimic the appearance of sickle cell disease-related ulcers. However, the rapid evolution of lesions, significant pain, ulcerated appearance with a yellow fibrinous base, and other histological elements should make one recognize that this is an important entity to keep in the differential diagnosis. This is true even if the patient has been adherent to hydroxyurea treatment for years. In addition, the association with hydroxyurea-induced leg ulcers may not be as strong as once thought, and biopsy has the potential utility of distinguishing between sickle cell disease ulcers, hydroxyurea-induced ulcers, and potential etiologies.
